# Supplementary figures and images for: SRGP-1/srGAP and AFD-1/afadin stabilize HMP-1/⍺-catenin at rosettes to seal internalization sites following gastrulation in C. elegans
Source: PLoS Genet. 2023 Mar 3;19(3):e1010507. doi: 10.1371/journal.pgen.1010507 (PMC10016700; doi:10.1371/journal.pgen.1010507)

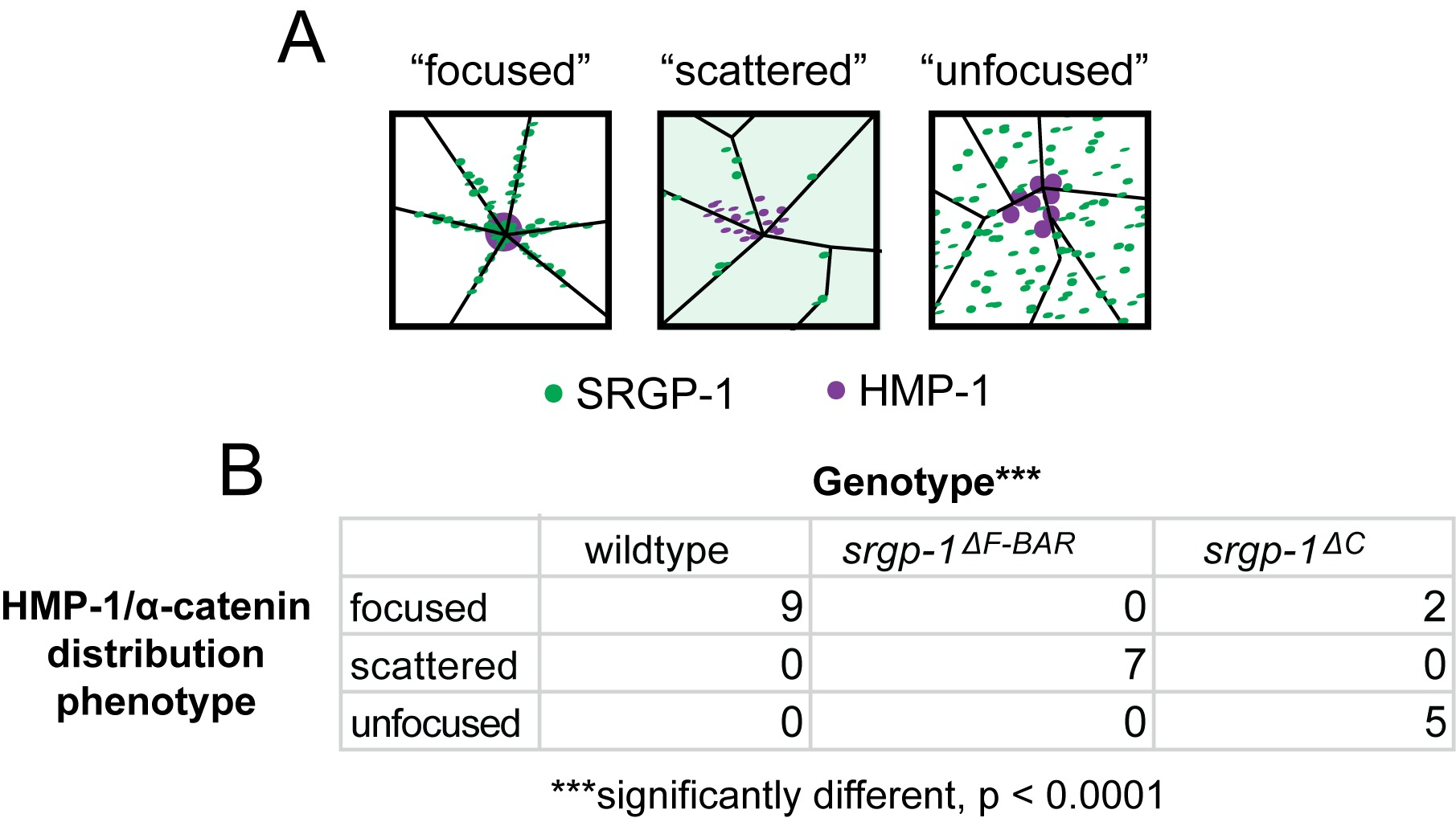

Supplement: S1 Fig — (A) Visual summary of the three basic patterns of SRGP-1 and HMP-1 localization based on Fig 3. (B) Incidence of HMP-1 localization defects in wildtype, srgp-1ΔF-BAR, and srgp-1ΔC mutants. ***, p < 0.001 (Chi-square analysis). (TIF) [file pgen.1010507.s001.tif]

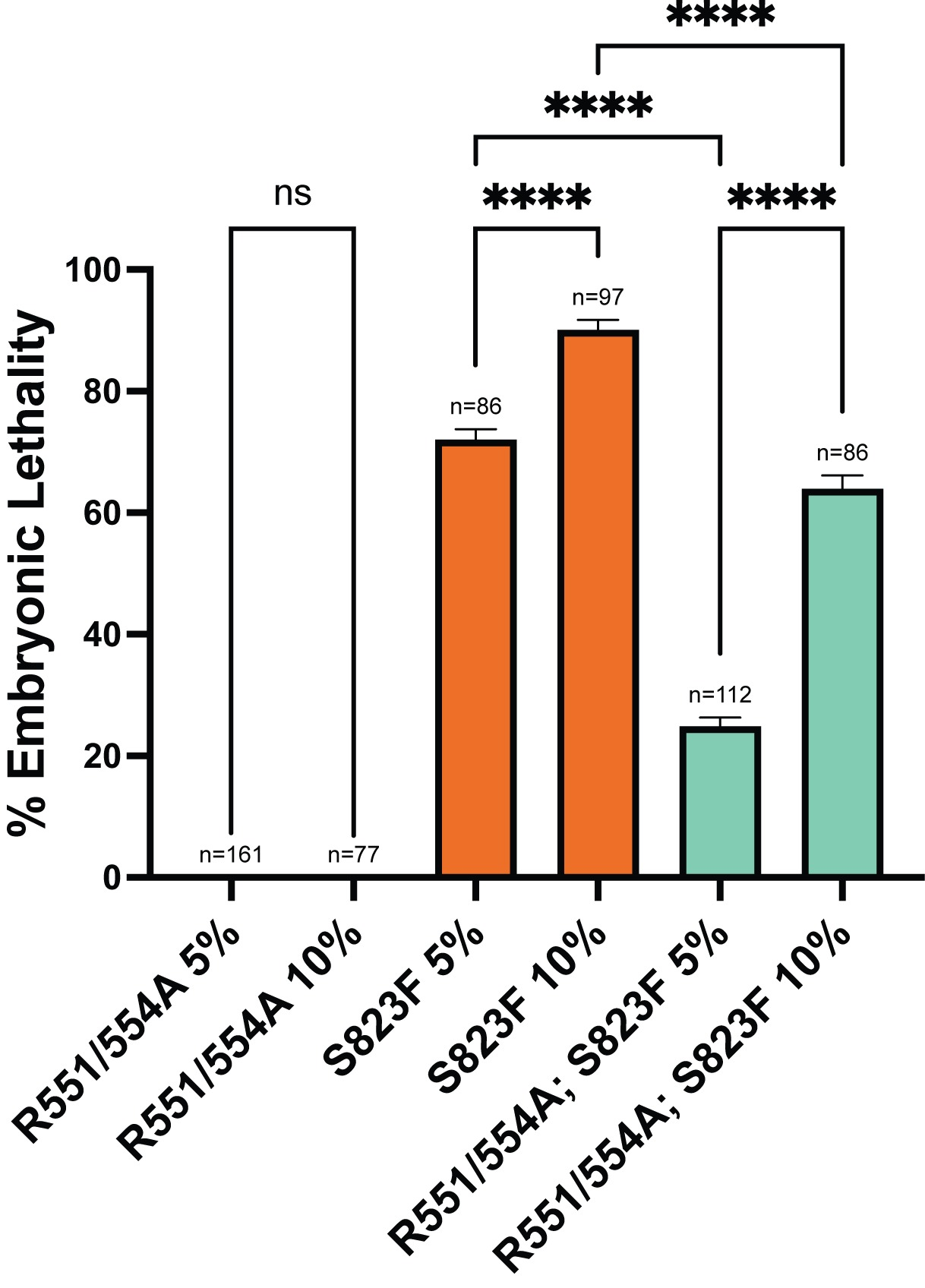

Supplement: S2 Fig — Embryonic lethality of hmp-1R551/554A, hmp-1S823F, and hmp-1R551/554A;S823F homozygotes when mounted on pads of 5% or 10% agar. (TIF) [file pgen.1010507.s002.tif]

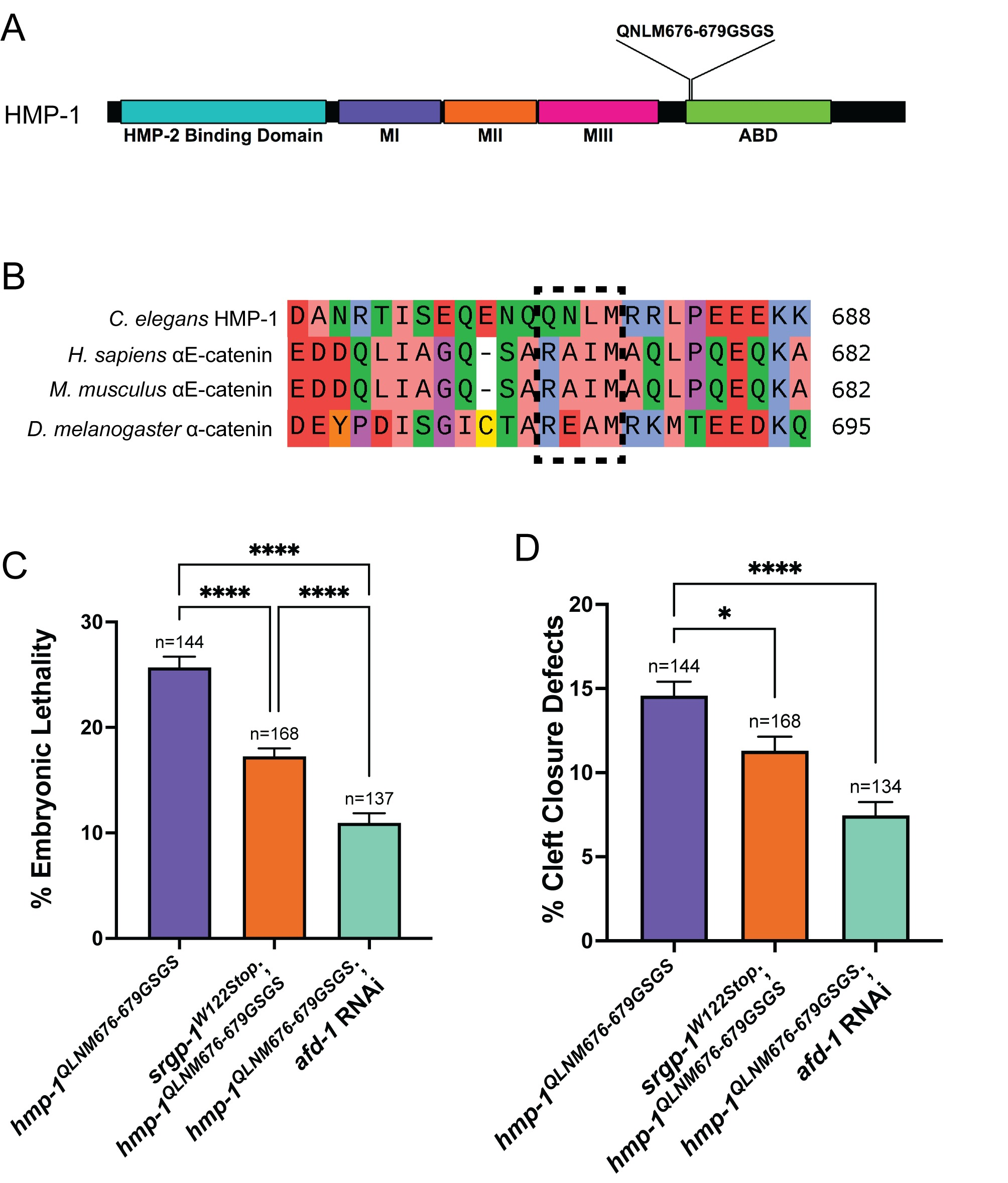

Supplement: S3 Fig — (A) A domain map of HMP-1 showing the location of the QNLM676-679GSGS mutation. (B) A MUSCLE alignment of C. elegans, human, mouse, and Drosophila ⍺-catenin at the RAIM site that was converted to GSGS by Ishiyama et al. (2018). Residues that have similar properties are assigned the same color. (C) Embryonic lethality on DIC mounts. (D) Percentage of cleft closure defects observed on DIC mounts. ****, p < 0.0001; *, p < 0.05. (TIF) [file pgen.1010507.s003.tif]

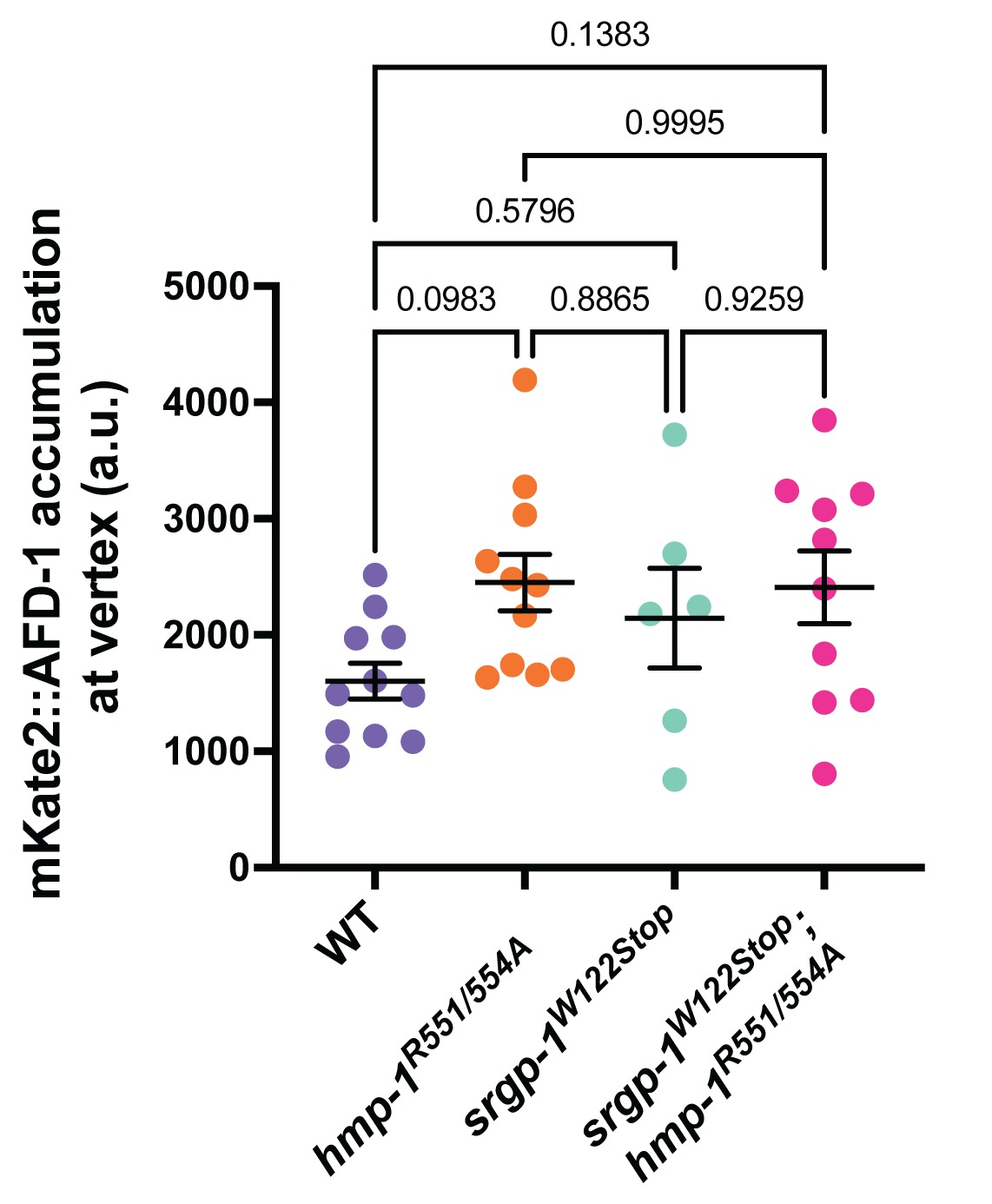

Supplement: S4 Fig — A graph depicting total accumulation of mKate2::AFD-1 at the vertex of the anterior rosette. hmp-1R551/554A mutant backgrounds show an increase of AFD-1 accumulation, however none of the differences between groups rise to the level of statistical significance. p-values are indicated. (TIF) [file pgen.1010507.s004.tif]
