# Supplementary material for: SRGP-1/srGAP and AFD-1/afadin stabilize HMP-1/⍺-catenin at rosettes to seal internalization sites following gastrulation in C. elegans
Source: PLoS Genet. 2023 Mar 3;19(3):e1010507. doi: 10.1371/journal.pgen.1010507 (PMC10016700; doi:10.1371/journal.pgen.1010507)
Supplement: S1 Table — (DOCX) [file pgen.1010507.s005.docx]

| Strain | Genotype |
| --- | --- |
| SU874 | *srgp-1(jc52[R536A])IV* |
| SU897 | *srgp-1(jc51[srgp-1::mNeonGreen::3xFlag + LoxP])IV; hmp-1(jc59[hmp-1::mScarlet-I + Lox511])V* |
| SU964 | *srgp-1(jc72[W122Stop])IV* |
| SU984 | *hmp-1(jc80[R551/554A])V* |
| SU985 | *hmp-1(jc81[HMP-1R551/554A::GFP + LoxP + unc-119(+) LoxP *cp20])V* |
| SU986 | *srgp-1(syb4178[srgp-1Δ750-1059::3xFlag::mNeonGreen])IV; hmp-1(jc58[hmp-1::mScarlet-I + Lox511])V* |
| PHX4178 | *srgp-1(syb4178[srgp-1Δ750-1059::3xFlag::mNeonGreen])IV* |
| SU992 | *srgp-1(jc85[srgp-1ΔF-BAR(57-337)::mNG + Lox2722 ])IV* |
| SU994 | *srgp-1(jc85[srgp-1ΔF-BAR(57-337)::mNG + Lox2722 ])IV; hmp-1(jc58[hmp-1::mScarlet-I + Lox511])V* |
| SU998 | *srgp-1(jc85[srgp-1ΔF-BAR(57-337)::mNG + Lox2722 ])IV; hmp-1(jc80[R551/554A])V* |
| SU1005 | *srgp-1(jc72[W122Stop])IV; hmp-1(jc80[R551/554A])V* |
| SU1004 | *srgp-1(syb4178[srgp-1Δ750-1059::3xFlag::mNeonGreen])IV; hmp-1(jc80[R551/554A])V* |
| SU1059 | *hmp-1(jc103[R551/554A, S823F *fe4])IV* |
| SU1102 | *hmp-1(fe4[S823F])V 6x outcross* |
| SU1067 | *hmp-1(jc107[QNLM676-679GSGS PAM mutated])V* |
| SU1074 | *afd-1(cp343[mKate2-C1::3xMyc::AFD-1 + Lox2272]) I; srgp-1(jc72[W122Stop])IV; hmp-1(cp20[HMP-1::GFP + LoxP + unc-119(+) LoxP])V* |
| SU1075 | *afd-1(cp343[mKate2-C1::3xMyc::AFD-1 + Lox2272]) I; srgp-1(jc72[W122Stop])IV; hmp-1(jc81[HMP-1R551/554A::GFP + LoxP + unc-119(+) LoxP *cp20])V* |
| SU1076 | *srgp-1(jc72[W122Stop])IV; hmp-1(jc107[QNLM676-679GSGS])V* |
| SU1069 | *afd-1(cp343[mKate2-C1::3xMyc::AFD-1 + Lox2272]) I ; hmp-1(jc81[HMP-1R551/554A::GFP + LoxP + unc-119(+) LoxP *cp20])V* |
| LP821 | *afd-1(cp343[mKate2-C1::3xMyc::AFD-1 + Lox2272]) I; hmp-1(cp20[HMP-1::GFP + LoxP + unc-119(+) LoxP])V* |
