# Supplementary material for: SRGP-1/srGAP and AFD-1/afadin stabilize HMP-1/⍺-catenin at rosettes to seal internalization sites following gastrulation in C. elegans
Source: PLoS Genet. 2023 Mar 3;19(3):e1010507. doi: 10.1371/journal.pgen.1010507 (PMC10016700; doi:10.1371/journal.pgen.1010507)
Supplement: S2 Table — (DOCX) [file pgen.1010507.s006.docx]

| Sequence Name | Sequence 5'-3' |
| --- | --- |
| guide for SRGP-1 C-terminal tag | agacgcagccaaaactaTCA |
| guide for SRGP-1 W122Stop | ACTGTACTGGAGGTGTGCTG |
| guide for SRGP-1^R563A^ | CCGATTGTGAACCAGAAACT |
| 5' guide for SRGP-1ΔF-BAR | AGCAGACAGAAGATGCTCAC |
| 3' guide for SRGP-1ΔF-BAR | TATCATCACCAAGTTGTGGA |
| guide for HMP-1^QNLM676-679GSGS^ | ATTCTGTTGATTTTCTTGTT |
| SRGP-1 C-terminal Tag 5' homology arm Forward | CACTGAGAGATCAGCTTCAGTTGATG |
| SRGP-1 C-terminal Tag 5' homology arm Reverse | GGCGACAAGCATCAGCCCA |
| SRGP-1 C-terminal Tag 3' homology arm Forward | TGAtagttttggctgcgtct |
| SRGP-1 C-terminal Tag 3' homology arm Reverse | ggattttgtaaccgaacacttcc |
| SRGP-1ΔF-BAR 5' homology arm Forward | ctgaaagctacgtccaccaaaccc |
| SRGP-1ΔF-BAR 5' homology arm Reverse | TTTAGCAGCACACTCCAAATCGTGTAC |
| SRGP-1ΔF-BAR 3' homology arm Forward | CAAAAATTCTTCGAGGCCAATCATCAATTGTTC |
| SRGP-1ΔF-BAR 3' homology arm Reverse | GTTTCCATTGAGAAGGAAGTGCTCG |
| SRGP-1^R563A^ repair template | ATCCGCCATTGCTTACCTCTCTCGATATTCTCTTCGTAATCAGGGATTATTCGCTGTTTCTGGTTCACAATCGGAAATTAATCGATTCAGAGAAGCTTATGAAAGA |
| SRGP-1^W122Stop^ repair template | CAAAAGTATAGCTCAAAAGCACAAATCAGAAAGAAGTCGACGAGAAGGTTGaCCACAaCAtACgagtAGTACAGTATGGCATACGTTAGTTGAACAGACGAAAG |
| HMP-1^QNLM676-679GSGS^ repair template | AAGATATCAATTTGTGCTTGAATTTTCTTTTTCTCTTCTTCTGGCAATCGTCTgcttccgcttccTTGATTTTCTTGTTCaGATATCGTACGATTAGCATCTGCATTTGCTGCACCAA |

Supplementary Table 2
