# Supplementary material for: SRGP-1/srGAP and AFD-1/afadin stabilize HMP-1/⍺-catenin at rosettes to seal internalization sites following gastrulation in C. elegans
Source: PLoS Genet. 2023 Mar 3;19(3):e1010507. doi: 10.1371/journal.pgen.1010507 (PMC10016700; doi:10.1371/journal.pgen.1010507)
Supplement: S3 Table — (DOCX) [file pgen.1010507.s007.docx]

| p-values | WT + control RNAi | WT + *afd-1* RNAi | HMP-1^R551/554A^ + control RNAi | HMP-1^R551/554A^ + *afd-1* RNAi | *srgp-1^W122Stop^*  + control RNAi | *srgp-1^W122Stop^*  + *afd-1* RNAi | *srgp-1^W122Stop^* ; HMP-1^R551/554A^ + control RNAi | *srgp-1^W122Stop^* ; HMP-1^R551/554A^ + *afd-1* RNAi |
| --- | --- | --- | --- | --- | --- | --- | --- | --- |
| *srgp-1^W122Stop^* ; HMP-1^R551/554A^ + *afd-1* RNAi | <0.0001 | <0.0001 | <0.0001 | <0.0001 | <0.0001 | <0.0001 | <0.0001 |  |
| *srgp-1^W122Stop^* ; HMP-1^R551/554A^ + control RNAi | 0.5204 | 0.9073 | >0.9999 | <0.0001 | 0.0062 | <0.0001 |  |  |
| *srgp-1^W122Stop^*  + *afd-1* RNAi | <0.0001 | <0.0001 | <0.0001 | <0.0001 | <0.0001 |  |  |  |
| *srgp-1^W122Stop^*  + control RNAi | <0.0001 | 0.2464 | 0.0139 | 0.8523 |  |  | n.s. | p>0.5 |
| HMP-1^R551/554A^ + *afd-1* RNAi | <0.0001 | 0.0057 | 0.0001 |  |  |  | *** | 0.05≥p>0.01 |
| HMP-1^R551/554A^ + control RNAi | 0.6401 | 0.9272 |  |  |  |  | **** | 0.01≥p>0.001 |
| WT + *afd-1* RNAi | 0.0266 |  |  |  |  |  | ***** | 0.001≥p>0.0001 |
| WT + control RNAi |  |  |  |  |  |  | ****** | 0.0001≥p |
