# Supplementary material for: SRGP-1/srGAP and AFD-1/afadin stabilize HMP-1/⍺-catenin at rosettes to seal internalization sites following gastrulation in C. elegans
Source: PLoS Genet. 2023 Mar 3;19(3):e1010507. doi: 10.1371/journal.pgen.1010507 (PMC10016700; doi:10.1371/journal.pgen.1010507)
Supplement: Attachment — Submitted filename: response_to_reviewers_final_v2.docx [file pgen.1010507.s009.docx]

**Response to Reviewers**

Reviewer #1

1. The cellular phenotype of srgp-1. The formation of "regular" cell contact instead of rosette could be resulted from less effective coupling between the internalizing cells and the surrounding cells and therefore less efficient extension of the surrounding cells. The authors discussed the role of SRGP-1 in sustaining curved membrane -- such curved membrane/tip not only exist in a rosette but also prior to that (more obvious in earlier gastrulating cells when cells are bigger). In this case, the phenotype would provide insights on the adhesion with the internalizing cells, and enrich the "adhesion clutch" model for internalization. A phenotype that could be attributed purely to the new contact between the surrounding cells would be the torn-up/burst to expose the internalized cells after complete sealing. The Methods section did not mention the temporal resolution of the imaging, but if it is higher than the 5 to 10 minutes shown in the figures, it may be worth analyzing the phenotypes before complete sealing as well as to examine if there is torn-up after complete sealing.\
   a. We are uncertain if the Reviewer is referring to a specific figure. In our confocal movies, embryos were imaged for 50-60 minutes (longer often caused arrest) with 1-minute intervals. Many of the images in the figures are from the end of those hour-long movies, since acquisition started when it was most obvious the ventral cleft was about to seal (30-40 minutes before sealing). We could not image faster due to bleaching and photodamage, precluding analyses suggested by Reviewer 1. **We updated the text in lines 659 to clarify imaging methods.**
   b. The Reviewer also raises an important biological point: the rupture phenotype we observe could arise due to one or more non-mutually exclusive cellular mechanisms. Sealing failure could result from failure of surface cells to make new connections with one another, but rosette formation and sealing could also be fostered via maintenance of cadherin-dependent connections between surface cells and internalizing cells, tuned precisely so that internalization draws cells inward while promoting midline movement of cells remaining on the surface that are attached to the remaining stalk of surface connection of the internalizing cell. We believe adjudicating between these possibilities is a fascinating question for future studies. Unfortunately, current technology does not allow us to perform clean experiments to test the contributions of these mechanisms, which would require rescue of function in internalizing cells vs. those remaining on the surface. Instead, **we have included discussion of these possible mechanisms in the revised manuscript in lines 534-549.**
2. The cellular phenotypes of afd-1 loss of function. The manuscript offered the frequency of ventral closure defect, but did not provide the more detailed cellular phenotypes as in srgp-1. There may be some missed opportunity here: AFD-1 may function in the adhesion/coupling between the internalizing cells and the surrounding cells during internalization. The recent study in Drosophila germband extension suggests that Afadin/Canoe is recruited to rosette centers to cope with extremely high tension. Should AFD-1 function during internalization in addition to being at the rosette center, it would an important insight.
   a. AFD-1 is very dim on the ventral side of the embryo. It was barely detectable/measurable above background, except when it strongly accumulated at a rosette vertex. We could therefore not obtain convincing measurements for comparison to other regions of the ventral cleft or elsewhere. This limitation means that Reviewer 1 is correct: there could be other roles for AFD-1 during the final stage of gastrulation. In a separate paper in review at *PLoS Genetics*, our co-authors Mark Slabodnick and Bob Goldstein and their colleagues have investigated the role of AFD-1 earlier, during Ea/p internalization. They also find that AFD-1 is very difficult to image. We feel that analysis of Ea/p ingression is best left to this complementary paper and/or future analyses. **Our discussion in lines 534-549 addresses the coupling of internalization to midline movement of surface cells and our technical imaging limitations (see above).**
3. Regarding AFD-1 at rosette centers, the authors appear to favor the interpretation by the Drosophila study. Some of the experiments in the manuscript could provide a test. The cellular phenotypes of srgp-1 ("regular" cell contact) removes the extreme curvature associated with rosette centers. One would predict that AFD-1 localization would be reduced in these cases? Figure 6B examines AFD-1 localization in srgp-1 loss of function. However, the example image shows the least severe phenotype of srgp-1 where the rosette is there albeit with a less focused center.
   a. We are uncertain about Reviewer 1’s meaning here. In Figure 3, all SRGP-1 mutations result in a rosette that is less focused, with some disorganization of HMP-1, compared to WT. Figure 6B demonstrates that HMP-1 and AFD-1 aggregate into multiple puncta instead of a single aggregation site in these cases, so there is an effect on AFD-1 localization. Fig. S3A, however, shows that whatever functional effect this has, the quantitative effect on overall AFD-1 signal is subtle (see the green dots vs. the purple data points). Based on our genetic findings (see especially Fig. 5D and 6C), we believe that SRGP-1 and AFD-1 work largely separately to stabilize and organize HMP-1 at rosettes. We hypothesize that the two proteins act alongside HMP-1/α-catenin in different states of extension1.
4. The authors provide a nice discussion of related morphogenesis processes, such as basal extrusion and multicellular rosettes in convergent extension. It would be helpful to point out more explicitly that the multicellular rosettes in worm gastrulation involves a fundamentally different process (apical contraction in internalizing cells) than the multicellular rosettes in convergent extension (driven by edge contraction within a contiguous epithelium). The fact that the two mechanisms converge to the similar geometry of rosettes means that they could have shared aspects in terms of tension and adhesion. Making such a distinction would enhance the value of worm gastrulation as a model and thereby the value of the manuscript. Furthermore, the convergent extension type of rosettes also exist in the worm (ref 29 cited in the manuscript, convergent extension of the VNC). Making the distinction would help people be aware of the differences (and the complexity of worm morphogenesis). Finally, the authors mentioned rosettes in chick primitive streak. Some people attribute rosettes in vertebrate gastrulation to convergent extension. If the cited rosettes in chick is associated with cell internalization, it will be valuable to point that out and argue that these are worm gastrulation type of rosettes, and not the convergent extension type.
   a. We agree with this suggestion. **We have now added new text to further accentuate the differences between worm gastrulation and convergent extension in the Introduction (lines 182-189)**.

Reviewer #2

1) Non-C. elegans readers will find it challenging to follow the results based on the C. elegans-specific gene names, which are not always indicative of their function. For example, hmp-1 encodes HMP-1, which is the Ealpha-catenin homologue. Although they need to use gene names where appropriate, it would be helpful to refer to the protein in a more accessible way (e.g., HMP-1/Ce alpha-catenin).
   a. **We have tried to add the homologue names throughout using slash notation for HMP-1/α-catenin**, except where deletion and point mutant allele names made that cumbersome. In addition, **we have used slash notation in subheads and at the start of each paragraph for SRGP-1/srGAP and AFD-1/afadin.** Since these gene names sound like contractions of their vertebrate counterparts, we were less fastidious about using slash notation, thereby reducing verbiage.
2) The timing of gastrulation and internalization of Ea/Ep cells in relation to the cleft and the rosettes is not clear for the reader. For example, are the cells that are moving in while the rosettes form also part of the Ea/Ep internalization, or are these cells moving in after as part of the cell re-arrangement to close the cleft? All of the movies from Figure 2 onward have time=0 min when rosettes are clearly visible at the ventral surface. However, Figure 1 has time=0 min as ‘before last ingression’. Are these the same thing? What is ‘before last ingression’? If it means something else (the word before is confusing), they should be consistent with timing between figures. Also, adding a schematic to show the relative timing of all of these events would be helpful (e.g., internalization of Ea/Ep, cleft closure, rosette formation and resolution). Especially since we are only shown DIC movies for cleft closure phenotypes, but then are only shown fluorescence images for rosette phenotypes, this makes it confusing to interpret the timing of cleft closure defects in the mutants.
   a. We are studying events well after Ea/p internalization. Please see comments above regarding a separate paper analyzing Ea/p. However, we agree that it is important that readers are clear on this point. Our analysis is restricted to the end of gastrulation, i.e., the very last internalization events. We have made the following changes:
      i. **We have used the phrases “end of gastrulation” (multiple places) to refer to the cleft sealing event we have focused on, and “beginning of gastrulation” to refer to Ea/p ingression** **(line 54)** in the revision to improve the language to clarify this point. To further clarify this point, **we have added a timeline to Figure 1 (new Fig. 1A) that shows when the events we have analyzed occur relative to Ea/p internalization.**
      ii. **We have updated the text line 66 to reflect the time of cleft closure** relative to the Ea/Ep internalization events.
3) As a related comment, the rosettes appear to be quite large, covering a large portion of the ventral surface, while the cleft is relatively small. It is hard to see how minor perturbations in the rosettes (e.g., stability or curvature of cells) could cause such drastic effects on cleft closure. Are there adhesion defects between other 'non-rosette' ventral cells, or is this more strictly because of mechanical forces causing rupture?
   a. This is a difficult question to answer. The rosettes form at either end of the cleft. It is unclear if they are important for initiating adhesion between all cells in the ventral cleft or if there is some other indirect mechanical interplay involved. This is something we hope to explore in future studies, which require single-cell approaches that are currently technically challenging.
4) Is the w122stop allele a null? It would be good to clarify this for the reader.
   a. **We have updated the text to lines 155 to reflect that this allele functions as a null.**
5) Lines 135-140 are not clear as written. The authors indicate that there are cleft closure defects in embryos with the delta F-bar mutant allele, but then say that they are not significant (and is ns on their graph). They could change the wording to clarify that this means something given that there are no defects in control embryos. I imagine this is an issue with n’s.
   a. **We have updated lines 169-170 of the text to clarify our meaning here.**
6) The flow from the section describing Figure 3 to Figure 4 could be improved to better explain the rationale for those experiments. One way to do this is to better connect to the previous finding. For example, the authors show that sgrp-1 alleles are differentially required for hmp-1 localization, so mutating hmp-1 to make protein that is in an extended conformation could be predicted to override the requirement for srgp-1.
   a. This was exactly our rationale; we thank Reviewer 2 for this suggested phrasing. **We have added similar language to lines 232-234**:
7) Lines 183-184 are confusing as written. “.. as a result, the HMP-1 M domain adopts a constitutively open conformation that prevents the recruitment of the SRGP-1 C-terminus..” Why? I think the issue is that there is no description of the structural function of alpha-catenin (or HMP-1) in the introduction, so the reader is learning this information for the first time in the results. Similarly, the follow-up statements in lines 185-187 where they refer to how an extended conformation of alpha-catenin activates actin binding or recruitment of vinculin and afadin. How does this relate to HMP-1 structurally? I think it is an issue where information on the structural regulation of alpha-catenin needs to be provided to the reader earlier in the manuscript (introduction) to understand for the rationale behind making the salt-bridge mutants. They could keep a sentence in the results to remind the reader of this relevant information.
   a. We agree that we may have expected the reader to infer too much here. We are happy to add additional background to the Introduction regarding HMP-1 and the M domain as suggested so that readers are not blindsided by new information. **We have added this additional background in lines 124-133.**
8) Figure 4. Since the delta Fbar phenotypes are already weaker, it might be challenging to detect suppression with the double/triple mutants. I think it is an issue of being cautious of interpretation. For example, saying that the C-term mutant suppresses is fine, but I would say it is inconclusive for the delta Fbar mutant.
   a. **We appreciate Reviewer 2’s attention to detail here. We have updated the language in lines 247, 250-251 to indicate that we were unable to detect suppression in the case of ΔF-BAR.**
9) Figure 4C. The increase in egg-laying with the salt-bridge mutant is really interesting, and supports a change in mechanical properties in the spermatheca – e.g., actomyosin contractility, where srgp-1 is known to function. This is really interesting, and the authors could comment on this finding in the discussion.
   a. We believe Reviewer 3 is referring to our original paper on *srgp-1* (Zaidel-Bar et al, 2010. *J. Cell Biol.* **191**, 761-9), where we showed in a supplemental figure that SRGP-1 is expressed in the spermatheca. We are actually not aware that functional tests have shown a role for SRGP-1 in the spermatheca, but it is strongly expressed there. We agree that this result, coupled with the result Reviewer 2 mentions in the current paper is interesting, and we hope to investigate this in the future. **We have added text about the spermatheca (lines 277-281)**.
10) Lines 233-235. Again, more knowledge of the structural regulation of alpha-catenin and how it interacts with other regulators of F-actin and adhesion would be helpful to have in the introduction. It seems that the role of the M domain in regulating actin-binding is important, but is not described.
11) Figure 5B – The y-axis is called 'embryonic arrest' on the graph, but there is a ‘hatch’ category, which doesn’t make sense. Should this be % phenotype or another term that encompasses the different categories?
    a. **We changed the label on the Y-axis to “% Phenotype”**
12) Figure 5B, C – To help obtain more support for their model, did the authors try a double knockdown of both srgp-1 and afd-1 in the hmp-1 mutant? Or is the phenotype already at ‘max threshold’ with srgp-1 and hmp-1?
    a. We believe the latter is the case. Both *srpg-1* and *afd-1* were previously identified as potent enhancers of lethality in the *hmp-1* hypomorphic background, leading to nearly 100% embryonic lethality. Given this high lethality for each enhancer individually, we had concerns surrounding how to establish reliable conditions whereby very low-level knockdown of both *srgp-1* and *afd-1* was consistent from embryo to embryo.
13) Figure 5D – It is not clear why the authors did not also test the knockdown of both srgp-1 and afd-1 in the hmp-1 strain with both hypomorphic and salt-bridge mutants. Especially if they want to show that the suppression is alleviated by the loss of afd-1. I appreciate that the lethality increases with both knockdowns and the hmp-1 allele with the salt bridge mutants, but adding the hypomorphic mutation might give a clearer result.
    a. Our previous work showed that SRGP-1 binding to HMP-1 is abrogated when HMP-1 contains the salt bridge mutation (Serre et al., 2022. *Development* **149** (18): dev200775). Since both *srgp-1* and *afd-1* are strong enhancers of the *hmp-1* hypomorph and the *hmp-1* salt bridge mutation fails to suppress *afd-1* RNAi phenotypes, we would not expect *afd-1* RNAi to be suppressed in the *hmp-1^R551/554A;S823F^* background, and do not feel that this experiment is especially informative.
14) Figure 6. To further address mechanism, the authors should consider looking at differences in F-actin (and myosin) localization/enrichment in/near the anterior vs posterior rosettes. They could also see how this localization changes when HMP-1 with the salt-bridge mutants is enriched. This could help address the mechanical aspects of their model. For example, it isn’t clear if the salt-bridge mutants directly lead to an increase in actin-binding/crosslinking. Have the authors considered reducing myosin contractility via mild let-502 RNAi to reduce tension, and see how this affects localization of the HMP-1 salt-bridge mutants and/or AFD-1 at the anterior rosette?
    a. This is a very good idea, but very challenging technically. We have extensive experience with trying weak *let-502* RNAi based on a separate, recently published paper on *srgp-1* and *hmp-1* during embryonic elongation (Serre et al., 2022. *Development* **149** (18): dev200775). Obtaining convincing weak knockdown has proven challenging, especially when layered onto other loss-of-function genetic backgrounds. We note that in this previous paper we do show that the salt bridge mutation rescues a hypomorphic allele in the actin-binding domain of HMP-1. This suggests that among other things, constitutively opening the M domain does positively affect actin binding. **We infer that that the salt bridge mutant improves mechanical resiliency in the current paper using the proxy of mechanical pressure using different concentrations of agar in embryo mounts, which we highlight in the text more clearly in lines 270-277 (see Fig. S1).** Another technical challenge, at least in our hands, is that the CRISPR-tagged NMY-2 lines have significant early lethality; we worry that analyzing these lines, when combined with loss of function for other genes, will involve artifactual synthetic lethality.
15) In the discussion, the authors refer to the stabilization of HMP-1 in the context of its role in adhesion, but given that there is also a mechanical aspect to the CCC, it would be good to know how these would work in concert. Especially since there is some in vitro data suggesting that alpha-catenin binds mutually exclusively to E-cadherin or actin.
    a. We absolutely agree that there is a “mechanical aspect to the CCC”, if by this Reviewer 2 means that the CCC is a mechanosensor. This has been well documented in many systems. We are confused, however, by this request by Reviewer 2. We believe Reviewer 2 are referring to work from the Nelson/Weis group on αE-catenin, which suggested that homodimeric mammalian αE-catenin has higher affinity for F-actin than when αE-catenin is engaged (via β-catenin) with the cadherin complex. Not all α-catenins show such constitutive dimerization behavior or allosteric control of α-catenin/F-actin binding, including *Drosophila* α-catenin (Ishiyama et al., 2018. *Nat Commun* **9** (1), 5121) and our own work (actually carried out with the Nelson/Weis groups) on HMP-1 (e.g., Kang et al, 2017. *J. Biol. Chem.***29**, 7077-7086).
16) In the discussion, there is an emphasis on the role of SRGP-1 in controlling the curvature of the membrane of the cells in the rosette, but this was not shown or quantified in the results. The cell segment software shows the change in cell position and shape in 2D, but this statement refers to a parameter that requires measurements in 3D. For example, I don’t see the ‘blunted ends’ that the authors refer to in the srgp-1 mutant. To support their interpretation, they should show some 3D rendering and/or quantify differences. Or they could decrease this aspect of their discussion.
    a. 3d curvature measurements in this system would be very challenging. We were only referring to curvature in 2d (i.e., the cell tip captured in a small Z slice at the surface). We did show this visually in Fig. 3: see Fig. 3A vs. B at 5 min. Given available fluorophores and strains and the time allotted by the editors for revisions, we have thus far not been able to introduce a membrane marker for performing these measurements. As Reviewer 2 suggests, **we have reduced the discussion of this point to read as follows** (lines **471-477**; ~~blue strikethrough~~ indicates deleted text; **red bold** indicates added text):
       “A distinct role for the SRGP-1/srGAP N terminus is also suggested by our results. When the SRGP-1 F-BAR domain is deleted the tips of cells in the rosette are blunted **(see Fig. 3A vs. B at 5 min)** and HMP-1/⍺-catenin forms multiple aggregates in cells in the rosette, corresponding to less robust rosettes. ~~In our previous work we observed reduced membrane curvature at the leading edge during ventral epidermal enclosure, suggesting that SRGP-1 promotes highly curved membranes {Zaidel-Bar, 2010 #52}. Our present results are consistent with a similar role at nascent rosette vertices, which require that the plasma membranes of cell tips in the rosette adopt a high degree of curvature.~~ SRGP-1 may either stabilize ~~such~~ highly curved regions of the plasma membrane **at rosettes** or be recruited to such sites**, thereby stabilizing HMP-1/α-catenin. Future experiments will be needed to distinguish these possibilities.** ~~, leading to clustering of HMP-1/⍺-catenin at such sites to stabilize nascent adhesions. The loss of normal HMP-1/⍺-catenin accumulation at cell tips in~~ *~~hmp-1~~^~~ΔF-BAR~~^* ~~mutants is consistent with this possibility and suggests that not only is membrane curvature adversely affected, but that HMP-1/⍺-catenin recruitment to sites of high membrane curvature is reduced, with adverse effects on rosettes.”~~

Reviewer #3:

1) Fig 2. In legend correct what the white dashed line indicates (VC?) and change “White dotted lines outline… rosettes” to “Yellow dotted lined…”. Also, consider stating that HMP-1 also appears to accumulate to some extent at the posterior rosette vertex.
   a. **We updated the figure legend to make the suggested changes.**
2) Fig 3. Reading through Fig 3 several points of minor confusion arose. (i) Fig 1 showed persistent clefts in the F-BAR and CT deletions, but these are not indicated/present in the embryos in Fig 3. (ii) Both mutants seem to show defects in the accumulation of HMP-1 at the vertex, but the text concludes that this a function of the CT. (iii) Likewise both mutants seem to have issues forming a vertex. (iv) Maybe include a statement about effect of F-BAR on SFGP-1 localization, as both mutants seem to affect this as well. (v) The phenotype of F-BAR was considerably weaker (ns) in Fig 1 but in Fig 3 both mutants seem to show similarly severe phenotypes. Obviously, this is going simply by what’s shown in Fig 3. So perhaps semi-quantitative statements in the text could be helpful such as, “Vertices failed coalesce in A out of B F-BAR mutants vs C out of D CT mutants.”
   a. We thank Reviewer 3 suggesting places where we could have been clearer in the text. We wish to make several points:
      i. Constraints of confocal imaging did not allow us to image as many embryos as we were able to for our DIC analysis. Due to the low penetrance of cleft closure defects in these lines, none of the embryos we imaged (n is 7-8 each for 3A, 3B, and 3C) exhibited cleft closure defects, and the window of time we focused on (rosette formation and resolution) occurred after the left and right sides of the cleft had moved close enough together to make the cleft indiscernible. While the cleft is not visible in these images, they nevertheless demonstrate meaningful changes in localization patterns and cellular rearrangements.
      ii. We have previously demonstrated that the C-terminus of SRGP-1 is able to interact with the HMP-1 M domain (Serre et al, 2022. *Development* **149** (18): dev200775). The C-terminus in the ΔF-BAR background is present and presumably capable of interacting with HMP-1, but the F-BAR domain, which affects SRGP-1 localization and organization, is no longer present. In the ΔC background, SRGP-1 loses this functional interaction between HMP-1 and the C terminus. For these reasons, we suggest that the C-terminus is also affecting localization.
      iii. Our preferred view is therefore that the differential localization patterns of HMP-1 between the two SRGP-1 mutant backgrounds reflects effects on the arrangement and persistence of the rosette and its vertex.
      iv. We have made the following changes to the manuscript:
          1. We now include a statement about effect of ΔF-BAR on SRGP-1 localization, since both ΔF-BAR and ΔC affect SRGP-1 localization, and we have performed the semi-quantitative analysis Reviewer 3 suggests. **We have added that information in lines 215-224 and in new Supplemental Figure 1.**
3) Fig S1. Might it make sense to include statistics for the comparison of hmp-1(S823F) vs hmp-1(R551/554A; S823F) – both 5% and 10% - to show that R551/554A is suppressing?
   a. These comparison bars have been added.
4) Fig 5. The interpretation of the genetic data wasn’t terribly straightforward and could use some clarification and interpretation. Along those lines, statistics for the comparisons of WT + afd vs hmp-1(R551/554A) + afd; srgp-1 + control vs srgp-1; hmp-1(R551/554A) + control; srgp-1 + afd vs srgp-1; hmp-1(R551/554A) + afd would be helpful/necessary. Without these comparisons statements such as “there may be additional factors beyond AFD-1 that may stabilize HMP-1 when it adopts an open conformation” may be hard to make. There is also the general conundrum is that hmp-1(R551/554A) seems to suppress in some cases (srgp-1 + control and srgp-1 + afd) and enhance in another (WT + afd). What does this mean exactly?
   a. **We generated a matrix in the raw data file and as a supplementary table showing the p-values of all pairwise comparisons for Figure 5D.** We hesitated to add more comparison bars to the graph as we worried this would affect readability.
   b. **We added some text (lines 322-330) to more clearly explain the genetic interactions that we observed.**
5) Fig S2. Again, most readers will need better clarification and interpretation of the genetic data, such as the finding that srgp-1 and afd show suppression effects for the hmp-1(QLNM) mutant whereas in Fig 5 with hmp-1(R551/554A), srgp-1 and afd were showing enhancement effects. Along those lines, cell biological data accompanies the genetic data for the various mutants except for the hmp-1(QLNM) mutant. Could this be informative?
   a. The QNLM lesion was not tagged with a fluorophore, which precluded us from pursuing a more in-depth cellular/biological analysis.
6) Fig 6. (i) The specific questions being addressed by the experiments in Fig 6B were unclear. (ii) Fig 6/D were not referenced in the Results and the meaning/difference between C/D was not spelled out. (ii) The image of HMP-1 + afd looks very similar to WT, whereas the data in C/D suggest a major difference.
   a. We thank Reviewer 3 for catching this! **We have now added a reference to Fig. 6D and added additional text that we hope clarifies what the experiment shows (lines 349-351).** The difference is quantitative, based on measurements of HMP-1::GFP “nuggets”. We have found that the eye tends to assess “on/off” well in this particular case, but not quantitative differences.
7) Fig S3. It’s stated that “we could not perform the converse experiment to address whether AFD-1 recruitment requires HMP-1 at this stage of development” but S3B indicates hmp-1(RNAi). Is this correct?
   a. We erroneously included Fig. S3B in our original submission. In our hands, *hmp-1* RNAi conditions that allowed for early gastrulation events led to greatly decreased, but detectable, amounts of HMP-1::GFP in the embryo. Original Fig. S3B really then functions as a negative control: AFD-1 localization persists when HMP-1 is still present. This result is not that informative, so **we have deleted this panel from Fig. S3** in the resubmission. We include it here for reference:

Original Fig. S3B

      Use of a null allele is precluded by maternally deposited protein from the heterozygous mother. We have not yet managed to generate a degron allele of *hmp-1* that would specifically remove it in specific cell type at this stage. Promoter options for this experiment are currently very limited and so a proper version of this experiment awaits future technical developments.
8) Discussion. The figure supporting models in the discussion (Fig 7) wasn’t hugely informative. Points brought up during the discussion could potentially more fleshed out in this figure, which would also help the reader to frame the data’s meaning and significance.
   a. We agree that the Fig. 7C provides modest insight. We think the visual summary in Fig, 7A,B is useful, and we propose to retain it. In lieu of original Fig. 7C, we propose to add one mechanistic proposal, which will require substantial additional experiments beyond the scope of this work to test. An interesting model (one of several) is that SRGP-1 helps to stabilize membranes during the early phase of HMP-1 recruitment. In addition, based on other work recently published from our group (Serre et al, 2022. *Development* **149** (18): dev200775) SRGP-1 may stabilize an intermediate conformation of the HMP-1 M domain that normally promotes transition to an extended M domain. Once HMP-1 is extended, AFD-1 could then “stabilize” HMP-1, either through direct binding or by strengthening the HMP-1-associated actin network. The early phase (involving SRGP-1) is not constitutively necessary, however, since SRGP-1 loss does not lead to complete failure. Perhaps a subpopulation of HMP-1, due to thermal fluctuation or some other mechanism, can adopt the extended state without the presence of SRGP-1, but SRGP-1 “greases the skids”. **We have added a new Fig. 7B and related text in the body of the manuscript and the legend accordingly.**
9) Update reference 35
   a. **This change has been made.**
10) Consider changing “conferred” to “caused” on line 218
    a. **This change has been made (line 276).**
